# Single nucleotide polymorphisms in C-reactive protein (CRP) predict response to adjunctive celecoxib treatment of resistant bipolar depression

**DOI:** 10.1016/j.bbih.2023.100625

**Published:** 2023-04-20

**Authors:** Angelos Halaris, Daniel Hain, Rebecca Law, Lisa Brown, David Lewis, Maria Filip

**Affiliations:** a Loyola University School of Medicine and Loyola University Medical Center, 2160 South First Ave., Maywood, IL, 60153, USA; b Myriad Neuroscience, 6960 Cintas Blvd, Mason, OH, 45040, USA; c Department of Adult Psychiatry Medical University of Lodz, Aleksandrowska 159, 91-229, Lodz, Poland; d The Polish National Agency for Academic Exchange, Polna 40, 00-635, Warsaw, Poland

**Keywords:** Bipolar depression, Treatment resistance, Escitalopram, Celecoxib, C-reactive protein, Single nucleotide polymorphism

## Abstract

**Background:**

Affective illness has been associated with a proinflammatory state, and it is generally accepted that the immune system plays a key role in the pathophysiology of mood disorders. Since inflammatory biomarkers are elevated in bipolar disorder, anti-inflammatory combination therapies may enhance response and reverse treatment resistance.

**Purpose:**

In the present study we investigated the possible impact of single nucleotide polymorphisms (SNPs) within the CRP gene on CRP blood levels, treatment response and level-of-stress perception in our cohort of treatment-resistant bipolar-depressed patients receiving escitalopram and celecoxib, or escitalopram and placebo, as previously reported (Halaris et al., 2020).

**Methods:**

Study design, clinical findings, and CRP blood levels have been reported previously (Halaris et al., 2020; Edberg et al., 2018). In this follow-up study we extracted DNA from blood cells collected at baseline. Genome-wide genotyping was performed for all subjects using the Infinium Multi-Ethnic Global-8 v1.0 Kit. Based on reports in the literature indicating possible associations with psychiatric conditions, ten previously reported *CRP* gene polymorphisms were evaluated in a preliminary analysis. We focused on rs3093059 and rs3093077 were in complete LD. Carriers were defined as those possessing at least one C allele for rs3093059, or at least one G allele for rs3093077. Additionally, we determined blood levels of the medications administered.

**Results:**

Non-carriers of rs3093059 and rs3093077 had significantly lower baseline CRP blood levels than carriers (p = 0.03). Increased rates of HAM-D17 response (p = 0.21) and remission (p = 0.13) and lower PSS-14 scores (p = 0.13) were observed in non-carriers among subjects receiving celecoxib but they did not reach statistical significance. When examining all subjects, nominally significant associations between carrier-status and remission (p = 0.04) and PSS-14 scores (p = 0.04) were observed after correcting for treatment arm. Non-carriers receiving celecoxib had the highest rates of response and remission, and the lowest stress scores.

**Conclusions:**

Carriers of the CRP SNPs may have higher baseline CRP levels, although non-carriers appear to benefit more from celecoxib co-therapy. Determination of the carrier status in conjunction with pretreatment blood CRP level measurement may contribute to personalized psychiatric practice, but replication of the present findings is needed.

## 1 Introduction

Bipolar Disorder (BD) is a multi-faceted and recurring lifelong psychiatric illness characterized by an alternating course of manic or hypomanic and depressive episodes. It is a heterogenous disease with symptoms spanning multiple domains of emotion and behavior, including changes in mood, anxiety, memory, anhedonia, optimism, sleep, energy, appetite, libido and psychomotor activity including suicidality. Cognitive dysfunction and compromised psychosocial function may persist indefinitely (Sachs et al., 2007; Torres et al., 2010; Malhi et al., 2007; Green, 2006). While BD I is marked by manic and depressive episodes, BD II consists predominantly of depressive episodes (BDD) and hypomanic episodes. A feature of BD, much like in major depressive disorder, is treatment resistance. Treatment resistant bipolar disorder depressed (TRBDD) is defined as “failure to reach sustained symptomatic remission for 8 consecutive weeks after two separate treatment trials, at adequate therapeutic doses and treatment exposure, with at least two recommended monotherapy treatments or at least one monotherapy treatment and another combination treatment” (Hidalgo-Mazzei et al., 2019). Although the biological basis of treatment-resistance has yet to be fully elucidated, at least in a subset of TRBDD subjects, an underlying metabolic and immunologic dysregulation has been postulated (Felger et al., 2020; Haroon et al., 2018). This is in line with the preponderance of studies investigating the role of inflammation in psychiatric illnesses, especially depression, over the past two decades (Badini et al., 2022; Zou et al., 2022). It is now generally accepted that the immune system plays a key role in the pathophysiology of mood disorders. Inflammatory biomarkers are reportedly elevated in BDD, therefore anti-inflammatory combination therapy may enhance response to treatment and reverse treatment resistance (Fond et al., 2014; Goldsmith et al., 2016; Miller and Raison, 2016), Felger (2018); Pereira et al. (2021).

Many patients diagnosed with stress-related mood disorders have increased levels of pro-inflammatory mediators, such as C-reactive protein (CRP), and pro-inflammatory cytokines, such as interleukin-6 (IL-6), consistent with immune system activation (Almeida et al., 2007; Dargél A. et al., 2015; Miller and Raison, 2016). It is thought that the worsening and progression of BD over time is due, among other factors, to sustained immune system activation and neuroinflammation (Dantzer, 2009). Our clinical trial, the preliminary results of which were published in 2014 (Halaris et al., 2014), was designed to test the hypothesis that modulation of inflammation by co-administration of CBX, a COX-2 inhibitor, together with escitalopram (ESC), a selective serotonin reuptake inhibitor (SSRI), would modulate the immune system's pro-inflammatory response and associated neuroinflammation, thereby reversing treatment resistance and possibly accelerating treatment response in TRBDD patients. We have previously reported our findings on CRP, an acute phase reactant, that has long been recognized as a non-specific inflammatory biomarker (Edberg et al., 2018). CRP is produced in the liver, and following tissue injury, it binds phosphocholine on the surface of dying cells and activates the complement system, which aids in the removal of pathogens that may be inciting the inflammatory response (Hurlimann et al., 1966; Pepys and Hirschfield, 2003).

The pro-inflammatory cytokine IL-6 induces the transcription of CRP, and elevated levels of IL-6 have been reported in patients with BD (Gabay and Kushner, 1999; Goldsmith et al., 2016; Kushner and Feldmann, 1978). Besides during tissue injury, CRP may also be correlated with cortisol and dehydroepiandrosterone (DHEA) during the stress response (Pariante and Lightman, 2008). When the stress response is chronically activated, such as with elevated body-mass index (BMI) and other chronic inflammatory states, such as chronic stress, systemic inflammation may be at least partially responsible for depressive symptoms (Visser et al., 1999). Patients with a depressive disorder, commonly a part of BD, have been shown to have increased peripheral blood levels of CRP (Topić et al., 2013). It is possible that an elevated level of CRP is a key contributor to treatment-resistant depression in at least some BDD patients. Accordingly, in designing this study, we sought to investigate whether reducing pro-inflammatory mediators, such as CRP, would result in an augmented therapeutic response to antidepressant treatment in patients diagnosed with TRBDD. We acknowledge that the interaction of depressive symptoms and inflammation remains under investigation and has not been definitively established*.*

We reported baseline elevations of CRP in our TRBDD patients compared to Healthy Control (HC) subjects (Edberg et al., 2018), which were consistent with the literature (Dickerson et al., 2013; Tsai et al., 2012). Therefore, CRP may prove to be a useful biomarker for TRBDD, although possible confounding variables must be addressed, including BMI and other factors which are known to influence CRP. With CBX treatment, CRP decreased significantly compared to placebo (PBO), indicating that inflammation is significantly reduced in CBX-treated patients and very likely responsible for the enhanced treatment outcome we observed. These results were corroborated by the positive correlation that emerged between the 17-item Hamilton Depression Rating Scale (HAM-D17) scores and CRP by week 8 (Edberg et al., 2018). Therefore, CRP may prove to be a useful biomarker for monitoring and possibly predicting treatment response during combined SSRI + CBX treatment.

The present study is an extension of the clinical study we published previously (Halaris et al., 2020). In view of significant findings we reported about CRP blood levels in our cohort of TRBDD patients (Edberg et al., 2018), we sought to investigate the possible impact of single nucleotide polymorphisms (SNPs) within the *CRP* gene on CRP blood levels, treatment response as quantitated with the 17-item Hamilton Depression Rating Scale (HAM-D17), and level of stress perception via the Perceived Stress Scale (PSS-14) in our cohort of TRBDD patients receiving ESC and CBX, or ESC and PBO, as previously reported (Halaris et al., 2020).

## 2 Methods

### 2.1 Study subjects

As described previously (Halaris et al., 2020), study participants had a diagnosis of BD I or BD II based on the Diagnostic and Statistical Manual of Mental Disorders IV (DSM-IV) at the time of screening, and had no other co-morbid medical diagnosis, or a co-morbid psychiatric diagnosis, with the exception of anxiety disorder. Participants had to score at least 18 on the HAM-D17 to be included. To classify as treatment resistant, participants had to have failed two or more adequate trials of antidepressant medications and/or a mood stabilizer or atypical antipsychotic medication. Treatment resistance was quantitated using the Maudsley Staging Method (MSM) as described by Fekadu et al. (2009). In this two-arm cohort, 27 subjects received escitalopram (ESC) in combination with CBX, while 20 subjects received ESC in conjunction with identically appearing placebo (PBO). However, 3 subjects (PBO) are missing genotype data for rs3093059 and rs3093077 and were excluded from this present study. Baseline CRP levels were available for 39 of the 44 subjects, and week 8 CRP levels were available for 42 of the 44 subjects.

### 2.2 Study design

The study was approved by the Institutional Review Board (IRB) of Loyola University and was conducted according to the principles of the Declaration of Helsinki. Subjects had to complete a written informed consent document following verbal explanation of the study by one of the study clinicians. This was a 10-week, randomized, double-blind, placebo-controlled, two-arm study of TRBDD patients who would receive either escitalopram (ESC), in combination with CBX, or ESC in combination with placebo (PBO). The study involved a minimum 2-week washout phase, a 1-week PBO run-in phase, and an 8-week treatment phase. Study subjects were evaluated at weeks 1, 2, 4, and 8 of treatment. Throughout the entire study, study subjects were maintained on a mood stabilizer (other than lithium) and/or an atypical antipsychotic, as required to achieve mood stabilization. If they were already receiving such a mood stabilizer, they were allowed to remain on it for the duration of the study. They had been receiving mood stabilizing medication for at least three months prior to study entry*.* This was to prevent switch to mania or hypomania thereby endangering the welfare of the patient and also misleading the investigators into falsely assuming that a bona fide enhanced antidepressant action of the CBX add-on was occurring. A total of 47 patients who met severity criteria (baseline HAM-D17 ≥ 18) were randomized to one of the two treatment arms and received treatment for 8 weeks. Baseline and week 8 CRP blood levels were collected. Rating scales (HAM-D17 and PSS-14) were administered at baseline and weeks 1, 2, 4, and 8. Response was defined as ≥ 50% reduction in HAM-D17 from baseline to week 8. Remission was defined as week 8 HAM-D17 total score ≤7. Complete PSS-14 scores were available for 33 of the 44 subjects.

### 2.3 Blood concentrations

Blood was drawn for inflammation biomarkers at baseline and week 8. Blood draws were scheduled between 9 and 10 a.m. to control for any diurnal variations. Whole blood was spun down and separated into either serum or plasma and stored at − 80 °C. Plasma samples were analyzed using Zymutest High Sensitivity CRP (*hs*CRP) enzyme-linked immunosorbent assay (ELISA) kit (Hyphen Biomed®, Neuville-sur-Oise, France). This is a highly sensitive “one step” sandwich ELISA technique specific for human CRP. Plasma levels of *hs*CRP are expressed as *μ*g/ml. Blood levels of cytokines were determined using a Randox Cytokine and Growth Factors High-Sensitivity Array assay (Randox®, London, UK).

### 2.4 Drug levels of celecoxib, escitalopram and metabolites

Serum concentrations of CBX, ESC, and ESC metabolites were measured in an effort to rule out changes in the serum concentration of ESC due to possible drug interaction with CBX or the accompanying mood stabilizer/antipsychotic that might interfere with the efficacy of this antidepressant drug. We used liquid chromatography mass spectrometry with the SCIEX QTRAP® 5500 LC-MS/MS System. ESC and desmethylescitalopram (DMESC) levels were available for 37 of the 44 subjects, and didesmethylescitalopram (DDMESC) levels were available for 27 of the 44 subjects. CBX levels were available for 19 of the 27 subjects receiving CBX.

### 2.5 Polymorphism determination

DNA was extracted from blood cells collected at baseline. Genome-wide genotyping was performed for all subjects using the Infinium Multi-Ethnic Global-8 v1.0 Kit. Based on reports in the literature indicating possible associations with psychiatric conditions, the following ten common CRP gene polymorphisms: rs3093059, rs3093077, rs2794521, rs3091244, rs3093062, rs2794520, rs1417938, rs1800947, rs1130864, and rs1205 were evaluated in a preliminary analysis. rs3093059 and rs3093077 were in complete LD. Carriers were defined as those possessing at least one C allele for rs3093059, or at least one G allele for rs3093077. Only SNPs rs3093059 and rs3093077 showed significant associations with response/remission rates. These SNPs were further analyzed as described below.

### 2.6 Statistical analysis

#### 2.6.1 Statistical analysis software

All statistical analyses were performed using R 3.5.1.

#### 2.6.2 Correction for multiple testing

No correction for multiple testing was performed in this exploratory study.

#### 2.6.3 Allele frequency analysis

Hardy-Weinberg equilibrium for rs3093059 and rs3093077 was established comparing the study data with the allele frequencies reported in BioSample: SAMN10492695 of the National Center for Biotechnology Information; no significant deviation in allele frequency was found.

#### 2.6.4 Treatment arm and HAM-D17 response and remission

Chi-squared test with Yate's continuity correction was used to assess the relationship between treatment arm and response or remission.

#### 2.6.5 Treatment arm and escitalopram levels

Welch's *t*-test was used to assess the relationship between escitalopram levels, as well as metabolite levels, and treatment arm.

#### 2.6.6 Celecoxib levels and HAM-D17 response and remission

Welch's *t*-test was used to assess the relationship between celecoxib levels and response or remission.

#### 2.6.7 CRP SNPs and CRP levels

Linear models describing the relationship between CRP SNP carrier-status, defined as subjects carrying at least one variant allele for rs3093059 and rs3093077, and baseline CRP levels and week 8 CRP levels in subjects receiving CBX adjunctive therapy or placebo, as well as all subjects combined, were assessed using Type II ANOVA.

#### 2.6.8 CRP SNPs, treatment arm, and HAM-D17 response and remission

Generalized linear models describing the relationship between CRP SNP carrier-status and response or remission in subjects receiving CBX adjunctive therapy were assessed using Type II ANOVA. When looking at all subjects combined, interactions between treatment arm and carrier-status were assessed first.

#### 2.6.9 CRP SNPs, treatment arm, and PSS-14 scores

Generalized linear models describing the relationship between CRP SNP carrier-status and PSS-14 scores over time in subjects receiving CBX adjunctive therapy were assessed using repeated measures ANOVA. When looking at all subjects combined, interactions between treatment arm and carrier-status were assessed first. The nlme package (version 3.1) in R was used to model the repeated measures.

#### 2.6.10 Covariates

All linear models included subject's body mass index, age, and sex as covariates. PSS-14 score analyses also included time as a covariate.

## 3 Results

### 3.1 Treatment arm and HAM-D17 response and remission

Subjects who received ESC in combination with CBX had significantly higher response (p = 0.03, OR [95% CI] = 5.00 [1.33,18.81]) and remission rates (p = 0.003, OR [95% CI] = 12.75 [2.40,67.69]) than those receiving antidepressant monotherapy (Table 1).

**Table 1:** Association of treatment group (CBX v non-CBX) with response or remission rates.

	Response	Non-Response	Remission	Non-Remission
Celecoxib	n = 21	n = 6	n = 17	n = 10
Placebo	n = 7	n = 10	n = 2	n = 15

OR = 5.00 [1.33,18.81] OR = 12.75 [2.40, 67.69].

p-value = 0.03 p-value = 0.003.

The odds ratios (OR's) of increased response or remission rates among those receiving celecoxib relative to placebo were determined**.**

### 3.2 Treatment arm and escitalopram levels

There was no difference in the week 8 blood levels of ESC (p = 0.22), DMESC (p = 0.48), or DDMESC (p = 0.64) between treatment arms (Table 2).

**Table 2:** Association of treatment group with week 8 blood levels of ESC and its metabolites, DMESC and DDMESC.

Week 8	ESC (ng/mL)	DMESC (ng/mL)	DDMESC (ng/mL)
Celecoxib (n = 22)	25.4 ± 19.3	11.7 ± 8.83	1.65 ± 1.25^A^
Placebo (n = 15)	33.0 ± 17.5	13.8 ± 8.86	1.46 ± 0.91^B^
P-value	0.22	0.48	0.64

A B.

n = 15; n = 12.

N = 5 subjects in the celecoxib arm and n = 2 subjects in the placebo arm were missing baseline ESC and DMESC measurements. N = 12 subjects in the celecoxib arm and n = 5 subjects in the placebo arm were missing baseline DDMESC measurements.

### 3.3 Celecoxib levels and HAM-D17 response and remission

Week 8 blood levels of CBX were not significantly different between responders and non-responders (p = 0.16), or remitters and non-remitters (p = 0.91) (Table 3).

**Table 3:** Relationship between response/remission and week 8 CBX blood levels. 8 subjects were missing week 8 celecoxib measurements.

Week 8	CBX (ng/mL)	p-value
Response (n = 17)	364 ± 270	0.16
Non-response (n = 2)	485 ± 62.2	
Remission (n = 15)	374 ± 287	0.91
Non-remission (n = 4)	385 ± 122	

CBX = celecoxib.

### 3.4 CRP SNPs and CRP levels

**Fig. 1:**
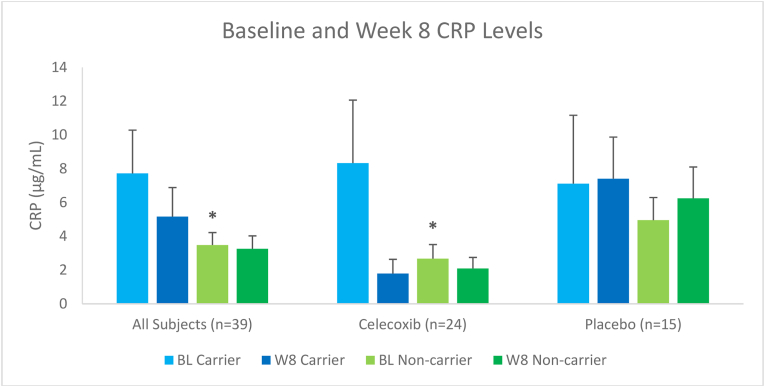
Baseline and Week 8 CRP levels among carriers and non-carriers in all subjects and in each treatment arm; shown as mean ± SEM. N = 3 subjects in the celecoxib arm and n = 2 subjects in the placebo arm were missing baseline CRP measurements. *p < 0.05 compared to CRP SNP carriers.

Baseline CRP levels were significantly increased amongst carriers of the *CRP* SNPs when compared to non-carriers in all subjects (p = 0.03) as well as in the CBX arm only (p = 0.03) (Fig. 1). No significant differences between carriers and non-carriers were observed when looking at week 8 CRP levels in all subjects (p = 0.39) (Fig. 1). BMI was a significant covariate in both of these models (p < 0.001), confirming many reports in the literature.

### 3.5 CRP SNPs, treatment arm, and HAM-D17 response and remission

**Fig. 2:**
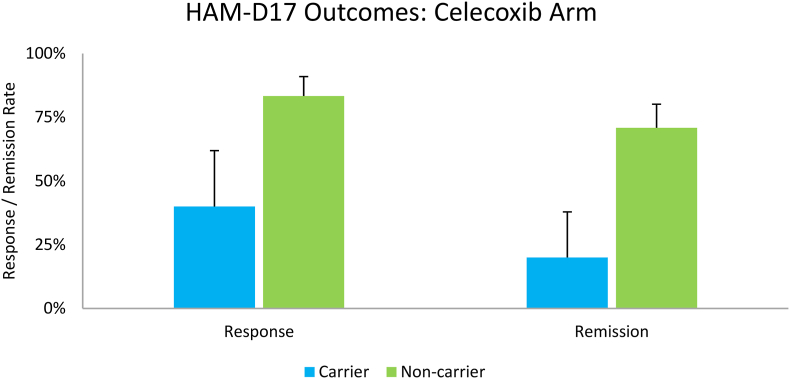
Response and remission rates among subjects on celecoxib co-therapy (n = 27) separated by CRP SNP carrier-status; shown as mean and ± SEP. No significant impact of carrier-status was observed.

**Fig. 3:**
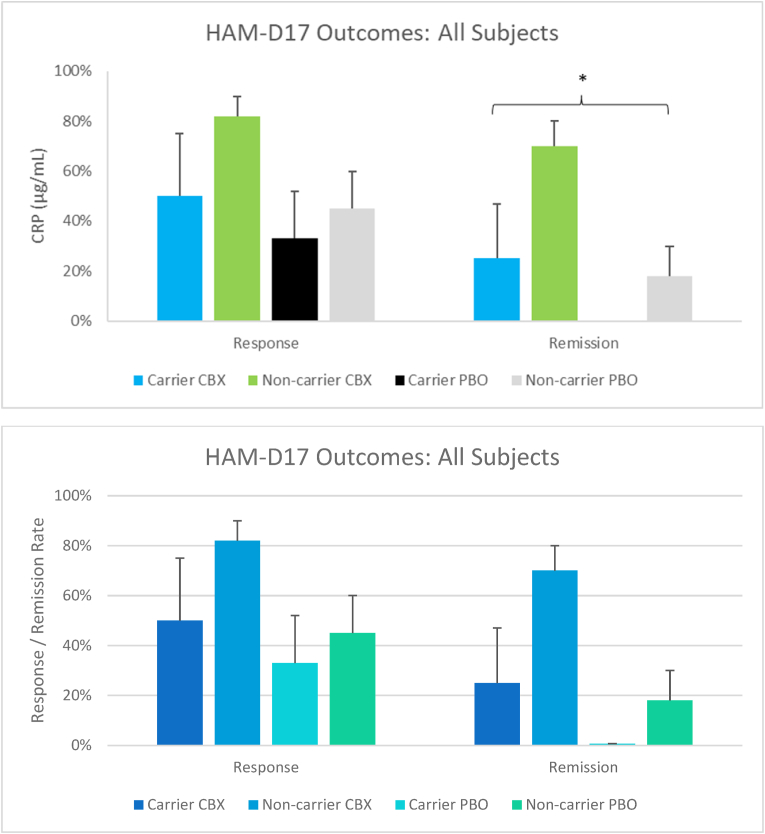
Response and remission rates among subjects on celecoxib adjunctive therapy (CBX; n = 27) or placebo combination (PBO; n = 17) separated by CRP SNP carrier-status; shown as mean and ± SEP. * significant main effect (p < 0.05) for carrier-status according to ANOVA after controlling for treatment group while also assessing interactions between carrier-status and treatment group.

The *CRP* SNPs, rs3093077 and rs3093059, were in complete LD (r^2^ = 1.0). Within the CBX arm, there was a non-significant trend for increased rates of response (p = 0.21) and remission (p = 0.13) among non-carriers (Fig. 2). When examining all subjects, after controlling for treatment arm, non-carriers had significantly increased rates of remission (p = 0.04) but not response (p = 0.22) (Fig. 3). No significant interactions were found between treatment arm and carrier-status. Non-carriers receiving CBX had the highest rates of response and remission.

### 3.6 CRP SNPs, treatment arm, and PSS-14 scores

**Fig. 4:**
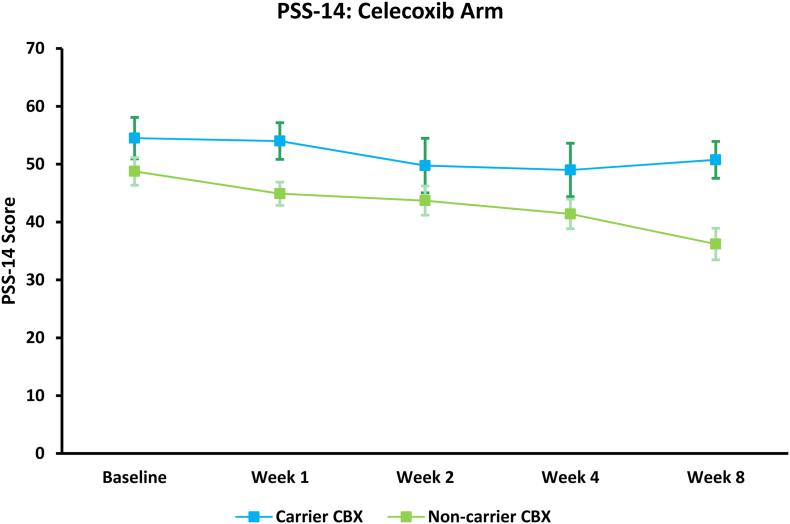
PSS-14 scores, among subjects with complete PSS-14 scores available, on celecoxib combination therapy (CBX; n = 21) therapy separated by CRP SNP carrier-status; shown as mean and ±SEM. No significant impact of carrier-status was observed.

**Fig. 5:**
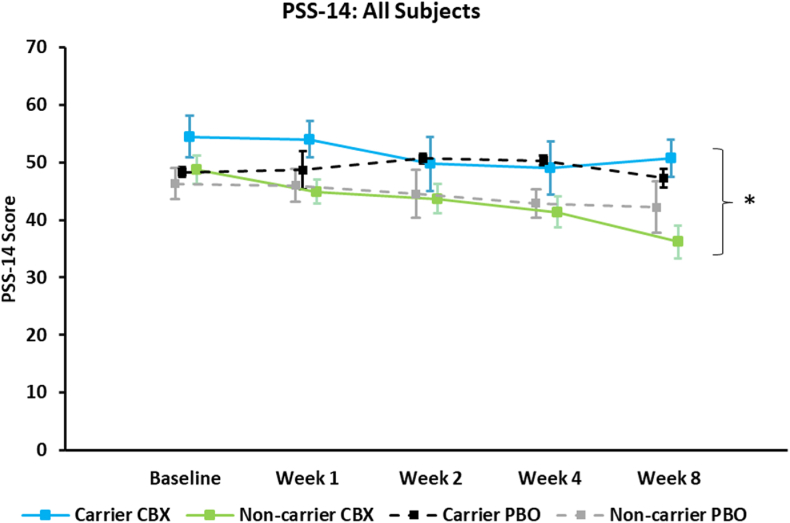
PSS-14 scores, among subjects with complete PSS-14 scores available, on celecoxib combination (CBX; n = 21) or placebo combination therapy (PBO; n = 12) separated by CRP SNP carrier-status; shown as mean and ±SEM. * significant main effect (p < 0.05) for carrier-status according to repeated measures ANOVA after controlling for treatment group and Week, while also assessing interactions between carrier-status and treatment group.

Within the CBX arm, PSS-14 scores were lower but non-significant (p = 0.13) among non-carriers (Fig. 4); however, increasing BMI was significantly associated with increasing stress scores (p = 0.01). When examining all subjects, after controlling for treatment arm, non-carriers had significantly lower PSS-14 scores (p = 0.04) (Fig. 5) and increasing BMI was non-significantly associated with increasing stress scores (p = 0.08). No significant interactions were found between treatment arm and carrier-status. Non-carriers receiving CBX had the lowest PSS-14 scores.

## 4 Discussion

Study subjects who received ESC in combination with CBX had significantly higher remission and response rates compared to those receiving antidepressant monotherapy. The CBX add-on not only significantly decreased depressive symptomatology, compared to PBO, but, additionally, subjects randomized to receive CBX had a significant antidepressant response at all time points (weeks 1, 4 and 8 of treatment) compared to the PBO recipients (Halaris et al., 2020). We conclude that CBX can be used adjunctively in patients with TRBDD to produce an enhanced and faster treatment response. Additionally, we previously demonstrated that blood levels of *hs*CRP are significantly higher in these patients compared to healthy controls (Edberg et al., 2018).

Cyclooxygenase-2 (COX-2) is an enzyme that is expressed in the central nervous system and plays an important functional role in the brain and periphery. COX-2 interacts with neurotransmitters, such as acetylcholine, 5-hydroxytryptamine and glutamate, but is also involved in the regulation of the central nervous system immune system and in inflammation via the effects of prostaglandins, in particular prostaglandin E2 (Gądek-Michalska et al., 2013; Bufalino et al., 2013; Famitafreshi and Karimian, 2020). The products of this enzyme play a major role as mediators of the inflammatory response and other medical states and cancer. Therefore, considering the emerging metabolic-immune basis of depressive illness, it has been hypothesized that pharmacologically induced modulation of inflammatory response may improve treatment response, reduce resistance and even arrest the neuroprogressive course of affective illness (Hosseini et al., 2014; Nery et al., 2008). In fact, a recent meta-analysis found that add-on therapy of anti-inflammatory drugs significantly improved depressive symptoms, response and remission among adults with major depressive disorder (Köhler-Forsberg et al., 2019).

Accordingly, adjunctive treatment with the COX-2 inhibitor, celecoxib (CBX), has been reported to lead to improved overall outcome in major depressive disorder (MDD) (Akhondzadeh et al., 2009; Abbasi et al., 2012; Hosseini et al., 2014; Müller et al., 2006; Fond et al., 2014; Müller, 2019). However, similar work in BDD is sparse (Ayorech et al., 2015; Arabzadeh et al., 2015; Husain et al., 2016; Nery et al., 2008), and none had directly addressed TRBDD until the present study was originally summarized in 2014 (Halaris et al., 2014). Our larger clinical study published in 2020, was the first to show that immune modulation using a standard antidepressant, escitalopram, adjunctively with CBX, is a safe and effective management of TRBDD (Halaris et al., 2020). Research at the molecular level should enable further characterization of the mechanism of action of COX-2 inhibition in effecting improved outcomes as well as the safety of such intervention.

When investigating the pathophysiologic relationship between depressive illness and inflammation with focus on CRP, the genetics underlying the expression of CRP become relevant. According to Pankow et al. (2001), the CRP blood level is as much as 40% heritable. The heritability of CRP expression has been documented in many studies with a variety of pathophysiologies, races and ethnic populations. It has also been reported in psychiatric disorders, notably repression (Ancelin et al., 2015; Su et al., 2009; Zhang et al., 2020)*.* If heritability can contribute to CRP blood levels, then the *CRP* gene and associated SNPs should be considered. The potential influence of *CRP* polymorphisms has been examined in non-psychiatric and psychiatric conditions. A host of non-psychiatric conditions have been examined and reported; they include conditions ranging from cardiovascular disease, cancer, stroke, sepsis, adiposity, COVID 2019, and others (Geng et al., 2016; Shangwei et al., 2017; Delongui et al., 2017; Shen et al., 2022; Tängdén et al., 2022; Huang et al., 2013). In regard to psychiatric disorders, there are a relatively limited number of studies, although the common outcome is confirmation of an association between CRP blood levels and SNPs, further differentiated between homozygotic vs heterozygotic alleles. Below we discuss intriguing and sometimes contradictory findings.

Here are a few reports addressing the *CRP* gene in depression and bipolar disorder. In a study with 990 participants at least 65 years of age, Ancelin et al. (2015) sought a possible association between variants in the *CRP* gene that influence expression of protein levels in depression. The minor alleles of rs1130864, rs1417938 and rs1800947 were associated with lower *hs*CRP levels compared with levels for men homozygous for the major alleles (defined as the most common allele of a SNP). In a cross-sectional study, Li et al. (2016) searched for a possible relationship between *CRP* SNPs, depressive symptoms and antidepressant efficacy. They examined 440 patients with first-episode depression and quantified depression using HAM-D17 at baseline and after 6 weeks. It was concluded that some *CRP* SNPs (rs1800947 and rs2794521) may be associated with depressive symptoms in a sex-specific manner. The rs2794521 SNP may be a predictor of antidepressant drug efficacy in females. Wang et al. (2018) examined 60 depressed patients with a family history of depression and 60 healthy control volunteers. CRP blood levels and the following SNPs were determined: rs3093059, rs1417938, 1800947, rs1130864 and rs1205. The results showed that patients with a positive family history for depression had a significantly higher circulating CRP level than controls. Yibulaiyin et al. (2017) investigated whether inherited CRP allelic variations may differ with respect to the presence of depressive symptoms. They screened 200 patients (100 MDD, with or without a family history of depression, and 100 healthy volunteers). The results showed that certain inherited CRP SNPs (A allele in rs1417938 and C allele in rs1205) could lead to up-regulation of serum CRP levels, and thus be associated with depression. Almeida et al. (2009) examined whether some polymorphisms of the CRP gene are associated with prevalent depression. They examined CRP SNPs – rs1130864 and rs1205 - in a population of 3700 men aged 70, or over. They concluded that the risk of depression was greater among people who carry the rs1205 G > A genetic polymorphism of the CRP gene. Evers et al. (2019) examined CRP concentrations in BD and established an association with genetic variants. Of 221 patients included in their study, 183 were genotyped for CRP SNPs. They concluded that low grade inflammation might play a role in mania and might be rather a state than a trait marker of bipolar disorder. Lastly, Boukouaci et al. (2018) suggested that CRP genetic diversity may contribute to the development of auto-immune comorbid disorders and rapid cycling, both proxy of BD severity. They concluded that such findings might predict complex clinical presentations of the disease leading to precision medicine in psychiatry. The two CRP SNPs we focused on in our study, rs3093059 and rs 3093077, have not been previously associated with bipolar disorder based on our literature search.

In view of apparent discrepancies in reported findings pertaining to both blood levels and CRP SNPs, brief mention about the CRP isomers is pertinent. CRP is present in at least two conformationally distinct forms: the native pentameric (pCRP) and the modified/monomeric (mCRP) isomer. These isomers are structurally unique from one another. Native C-reactive protein (nCRP) or pCRP is an oligo-protein and an acute phase reactant; its increased concentration in serum has been associated with chronic inflammatory disease. However, accumulating evidence suggests that pCRP possesses both pro- and anti-inflammatory properties (Wu et al., 2019; Melnikov et al., 2020). pCRP dissociates to form monomeric C-reactive protein (mCRP), a potent, short-lived, pro-inflammatory molecule that acts on endothelial cells, endothelial progenitor cells, leucocytes, and platelets, thereby amplifying inflammation (Wu et al., 2019; Rajab et al., 2020). Most of the research on CRP reported in the literature only refers to CRP itself, and often does not mention the isoforms or specify which isoforms were measured. Notably, *hs*CRP typically refers to the pentameric form (Li et al., 2016). However, a few laboratories have conducted studies to distinguish between each CRP isoform (Sproston and Ashworth, 2018). It is important that investigators seek to identify which polymorphisms are linked to BDD. The structure of CRP, namely the difference between the monomeric and pentameric forms of the protein, is a worthwhile area for future research. With mCRP possessing primarily proinflammatory properties, distinguishing between the concentration of mCRP and pCRP in a BDD patient may help explain response to treatment. We are not aware of any data showing associations of specific CRP SNPs with the CRP isomers. CRP levels, SNPs, and isomers may assist in guiding treatment selection for patients diagnosed with a depressive disorder.

Lastly, two observations derived from the above study data are worth commenting on. The first one relates to the observation that non-carriers in the CBX arm had better response and remission rates than carriers. These non-carriers did not show a significant change in the *h*sCRP blood level over eight weeks of CBX add-on treatment. Thus, the question arises, why did those patients who had “less inflammation” as reflected in lower blood levels of *hs*CRP, and did not have a significant reduction in *hs*CRP after receiving the CBX add-on, respond so well. The simplest answer could be the relatively small sample size in individual study cells. More likely explanations should be sought within the complex and interactive components of the body's inflammatory response. For example, the crosstalk between prostaglandins and cytokines (Yao and Narumiya, 2019); the role of TNFα in the anti-inflammatory response; the interaction between CRP and IL-6 (Giudice and Gangestad, 2018) extending to effects on indoleamine dioxygenase (IDO), a key enzyme regulating the diversion of tryptophan to the kynurenine pathway resulting in abnormal elevations of neurotoxic metabolites, notably quinolinic acid (QA). We have reported significantly higher baseline QA levels in the same sample of TRBDD study subjects compared to healthy control subjects (Castillo et al., 2020). Of note, Krause et al. have also reported an inverse relationship between QA and treatment outcome in unipolar depression demonstrating that high kynurenine predicted remission in patients with major depressive disorder receiving CBX add-on treatment (Krause et al., 2017).

Another observation pertains to stress perception by the individual, as reflected in their PSS scores. We found that in the overall sample, non-carriers had significantly lower PSS scores and non-carriers in the CBX arm tended to have lower scores as well. These findings raise the question about the “stress-inflammation” balance difference between carriers and non-carriers of the specific CRP SNP we analyzed in this study, since stress has been linked to inflammation in general, and specific inflammation markers including CRP. Numerous studies have investigated the relationship between PTSD and CRP and cytokines. Inflammation and SNPs in the CRP gene have been associated with increased blood CRP protein levels and illness severity in PTSD patients. However, the mechanism by which the CRP SNPs are involved in PTSD are unclear. A thorough discussion of this issue exceeds the scope and word limit of this article. However, we mention a study reporting on the association of CRP genetic variation with symptomatology, cognitive function, and circulating proinflammatory markers in civilian women with PTSD (Otsuka et al., 2021). The investigators reported that patients with the rs2794520 CC/CT genotype, compared to those with the TT genotype, showed significantly higher levels of *hs*CRP and *hs*TNFα., more severe PTSD symptoms, and poorer cognitive function. A two-way analysis of variance revealed a significant genotype-by-maltreatment interaction for more severe PTSD avoidance symptom.

In summary, the results of our study indicate the *CRP* SNPs were associated with significantly increased baseline CRP levels in all subjects (both the CBX & PBO groups together) and in the CBX subjects, but not the PBO group; likely an issue of power due to smaller sample size of the PBO group. Non-carriers receiving CBX had the highest rates of response and remission, and the lowest stress scores. Limitations of the current study include a small sample size, a 8-week treatment duration, and multiple testing. It is possible that the present findings may not be fully replicated without these limitations in a future study. Future studies should include *CRP* SNPs and further explore the relationship between carrier status, elevated blood levels of *hs*CRP and treatment response. Similarly, future studies should more precisely define whether adjunctive anti-inflammatory treatment will be beneficial independent of how significantly elevated blood CRP levels may be. Determining pentameric and monomeric CRP levels may help clarify this puzzle*.* Last, but not least*,* assessing specific symptoms in addition to overall illness severity has the potential to reveal meaningful associations about the individual patient, their symptom and genetic profiles and ultimately their response to treatment. Add-on anti-inflammatory treatment may be beneficial especially in patients with carrier status of specific *CRP* SNPs, as presented in this study.

## Role of the funding source

This study was supported by a research grant awarded to Dr. Angelos Halaris by the 10.13039/100007123Stanley Medical Research Institute (SMRI) (Stanley Foundation, Grant No. 10T-1401). The authors gratefully acknowledge the donation of celecoxib by 10.13039/100014476Pfizer Pharmaceuticals. Dr. Maria Filip was the recipient of a fellowship from the Polish National Agency for Academic Exchange.

## CRediT authorship contribution statement

Angelos Halaris conceived and designed the entire study, oversaw the data collection, the data analyses and the preparation of the manuscript. Daniel Hain and Rebecca Law conceived SNP analysis, performed statistical analyses, and analyzed the genetic data; they also reviewed and contributed to the preparation of the manuscript. David Lewis oversaw the data analyses and drug level determination and contributed to the preparation of the manuscript. Lisa Brown and Maria Filip contributed to the literature review and preparation of the manuscript.

## Declaration of competing interest

The authors report no conflict of interest.

## Data Availability

No data was used for the research described in the article.
